# Mind Wandering in a Multimodal Reading Setting: Behavior Analysis & Automatic Detection Using Eye-Tracking and an EDA Sensor

**DOI:** 10.3390/s20092546

**Published:** 2020-04-29

**Authors:** Iuliia Brishtel, Anam Ahmad Khan, Thomas Schmidt, Tilman Dingler, Shoya Ishimaru, Andreas Dengel

**Affiliations:** 1 German Research Center for Artificial Intelligence, Trippstadter Str. 122, 67663 Kaiserslautern, Germany; shoya.ishimaru@dfki.de (S.I.); andreas.dengel@dfki.de (A.D.); 2 TU Kaiserslautern, Department of Computer Science, Erwin-Schrödinger-Str. 57, 67663 Kaiserslautern, Germany; 3 School of Computing and Information Systems, University of Melbourne, Parkville VIC 3010, Australia; anamk@student.unimelb.edu.au (A.A.K.); tilman.dingler@unimelb.edu.au (T.D.); 4 TU Kaiserslautern, Center for Cognitive Science, Erwin-Schrödinger-Str. 57, 67663 Kaiserslautern, Germany; thomas.schmidt@sowi.uni-kl.de

**Keywords:** mind wandering, eye tracking, electrodermal activity, reading, attention-aware systems, meta-awareness

## Abstract

Mind wandering is a drift of attention away from the physical world and towards our thoughts and concerns. Mind wandering affects our cognitive state in ways that can foster creativity but hinder productivity. In the context of learning, mind wandering is primarily associated with lower performance. This study has two goals. First, we investigate the effects of text semantics and music on the frequency and type of mind wandering. Second, using eye-tracking and electrodermal features, we propose a novel technique for automatic, user-independent detection of mind wandering. We find that mind wandering was most frequent in texts for which readers had high expertise and that were combined with sad music. Furthermore, a significant increase in task-related thoughts was observed for texts for which readers had little prior knowledge. A Random Forest classification model yielded an $F_{1}$-Score of 0.78 when using only electrodermal features to detect mind wandering, of 0.80 when using only eye-movement features, and of 0.83 when using both. Our findings pave the way for building applications which automatically detect events of mind wandering during reading.

## 1. Introduction

The phenomenon of mind wandering is well known to all of us: While reading, driving, or engaging in a routine task, we often find our attentional focus drifting to internal thoughts or concerns. Mind wandering is defined as *“a shift in the contents of thoughts away from an ongoing task and/or external environment to self-generated thoughts and feelings”* [1]. Roughly, mind wandering can be classified along two dimensions: *task-related thoughts* (TRTs) and *task-unrelated thoughts* (TUTs). These types affect task performance and mental well-being of people differently. While *task-related thoughts* foster creativity and problem-solving ability [1,2], *task-unrelated thoughts* are associated with decreasing learning performance and reading comprehension [3,4].

One of the central challenges in mind wandering research is the complexity of detecting and quantifying it. Because of the covert and spontaneous nature of mind wandering [5], the main technique for its measurement is experience sampling (ES). ES is a methodology for assessing ongoing thoughts of participants performing a task [1,2]. The most established techniques are *self-caught* and *probe-caught* methods (for a comprehensive review see [6]). While the self-caught method depends on participants’ ability to monitor their thoughts, the probe-caught method can detect MW episodes the participant has overlooked. However, it can also be disruptive and even enforce unintentional episodes of mind wandering [6,7].

An alternative method for mind wandering detection is the use of physiological sensors. Complex and rather expensive techniques for mind wandering observation include fMRI [8,9,10] and EEG [2,11,12,13]. Simpler and more scalable detection can be accomplished using eye-trackers [4,14,15,16,17,18,19] and the measurement of electrodermal activity [20,21,22].

Research on mind wandering plays an important role in education where it is primarily associated with low task and learning performance [3,23,24]. Presumably, the attentional decoupling caused by mind wandering suppresses information processing from external sources (i.e., learning materials), thereby impairing learning performance. Moreover, the recognition of mind wandering is linked to meta-awareness–the ability to track and monitor one’s own thought process and subjective experience [25]. This ability strongly varies among learners. On the other hand, the shift of attention to internal thoughts can sometimes be required for a successful task performance [24]. Mental arithmetic, imagery [26] as well as autobiographical memory recall all require internally directed attention. This ambiguous role of mind wandering is often overlooked when developing methods to control learner’s attention and awareness.

Mind wandering detection and intervention provide new opportunities for *attention-aware learning systems*, in which the goal is to adapt to the mental state of learners [27,28]. Accurate detection of mind wandering onset is a challenging task [11,12,14], but recent studies showed the general feasibility of automatic mind wandering detection in learning tasks by using low-cost eye-trackers powered by machine-learning algorithms [14,27,29,30]. Maintaining attentional states by means of attention-aware technologies and adaptable presentation systems is supposed to support the learning process. However, there are some learning contexts where certain types of mind wandering may be desirable. A small number of studies investigating the effect of TRTs on learning performance indicated their critical role in learning at early stages where prior knowledge is low or absent [31]. In line with that, cognitive psychologists such as Faber et al. [32] identified the importance of contextual settings for the assessment of mind wandering.

To equip entire classrooms with attention-aware technologies, we seek low-cost and scalable techniques for detecting mind wandering. In this paper, we focus on reading as a crucial component of learning systems. Our work consists of two parts. The first part introduces a behavioral analysis and focuses on the effects of semantics and music on the frequency and content of mind wandering in a reading task. In the second part, we use sensory and behavioral data to build models to automatically detect episodes of mind wandering. Hence, our contribution is as follows:

- We investigate the role of semantic text relevance and music type on the frequency and type of mind wandering.
- We show the feasibility of using an EDA sensor as a single device for mind wandering detection, as well as a combination of EDA with eye-tracking. Compared to previous studies using the raw EDA signal as a neural marker of mind wandering, we use the method of convex optimization to decompose the raw EDA signal into underlying sub-components.

## 2. Triggers of Mind Wandering

Research in experimental psychology found strong evidence that interest [33], task difficulty, emotional state [1,9], monotone or highly atomized tasks [24], and distractibility [34,35] are strong determinants of mind wandering. Episodes of mind wandering can also arise from semantic stimulus processing if the stimulus evokes memories [32,36]. Smallwood et al. found that experience with a topic influenced the temporal focus of mind wandering [37]. Thus, people with a corresponding background reported significantly more frequent episodes of mind wandering. Faber and D’Mello investigated relationships between stimulus type and the content of mind wandering [32]. Their experiment with 88 participants showed that the content of mind wandering is widely spread across multiple thought categories and associated with various triggers. Semantically rich content was found to force mind wandering associated with memory retrieval. Beside semantics, music was also shown to influence the frequency and the content of mind wandering [9,38,39]. Taruffi et al. [9] showed that music triggering sad, low-arousal emotions was associated with more frequent episodes of mind wandering, compared with music triggering happy, high-arousal emotions. Feng and Bidelman [38]’s study investigated the relationship between music and mind wandering in the context of a lexical processing task and yielded similar findings. Their results showed that mind wandering occurred more frequently in conditions with unfamiliar music. The authors suggested that lower emotional arousal from unfamiliar music was linked to boredom, negative mood, and distractibility, resulting in an increased frequency of mind wandering. The nature and content of mind wandering is, therefore, complex and multifaceted, and the assessment of this mental state as merely detrimental to performance can be misleading. There is however, a lack of information pertaining to possible interaction effects between different contextual factors on mind wandering.

Based on the results from aforementioned studies, we hypothesize that texts perceived as highly relevant will cause more frequent episodes of mind wandering compared with less relevant texts. Additionally, we explore possible interaction effects between text types and music on mind wandering. Taking into account modern learning environments where the presence of background music during individual learning or reading is commonplace [39,40], one can realize the necessity to explore the interaction of several contextual factors on the content and frequency of mind wandering.

## 3. Indicators of Mind Wandering

In this section, we focus on two indicators of mind wandering: (1) eye movements and (2) electrodermal activity (EDA). In addition, we briefly introduce a theoretical framework for the physiology and functionality of EDA in the context of mind wandering research.

### 3.1. Mind Wandering and Eye Tracking

A strong relationship exists between the episodes of mind wandering and eye movement patterns [4,14,15,16,17,18,41]. In particular, fixations, blink rate, pupil diameter, eye vergence, and saccades were found to be strong indicators for the presence of mind wandering. Benedek et al., for instance, provided a comparative analysis of oculomotor behavior in conditions with internally (IDC) and externally (EDC) directed cognition using anagram and sentence generation tasks [42]. The results showed that IDC was associated with fewer and longer fixations, higher variability in pupil size diameter and eye vergence, and a lower angle of eye vergence. In a meditation task with a self-caught sampling method, Grandchamp et al. found a significantly smaller pupil diameter during episodes of mind wandering [16]. Smilek et al. investigated changes in a blink rate during mind wandering in a reading task using an auditory probe-caught method [18]. They found a higher blink frequency during mind wandering compared to the periods where participants were focused on a task. The aforementioned changes in oculomotor behavior may indicate attentional decoupling and the resulting suppression of the visual input within the episodes of mind wandering (for more details, see [25]).

Using findings from neuropsychology and applying machine-learning techniques, several research groups succeeded in building automated eye-based detectors of mind wandering. Bixler and D’Mello [14], for example, recruited 178 participants and asked them to read four texts and to report mind wandering using the self-caught method. Among other features, they used 40 global gaze features to build a user-independent mind wandering detector. The reported models achieved accuracy between 67% and 72%. Unfortunately, the authors used a high-end, expensive eye tracker, which restricts the deployment of their method on a large scale.

Addressing this issue, Hutt et al. [43] demonstrated the feasibility of using low-cost eye trackers for automatic mind wandering detection in a classroom setting. Using eye tracking data of 135 high school students recorded during computerized learning, their model achieved an $F_{1}$-Score of 0.59.

### 3.2. Mind Wandering and EDA

So far only a small number of studies used EDA as a neural marker for mind wandering detection. In addition to that, there is still a lack of theories explaining the link between episodes of mind wandering and alternations in EDA. In the following, we present a theoretical framework.

EDA (also skin conductance response (SCR) or galvanic skin response (GSR)) is associated with alternations in the electrical properties of the skin mediated by the level of physiologically induced sweating [44]. When the sudomotor nerves stimulate the production of sweat, the conductivity of the skin surface changes as a result of sweat secretion. Even though sweating primarily regulates body temperature, clinical studies investigating schizophrenia and assessment of pain pointed out a relationship between emotional arousal and electrodermal skin response [45]. There is considerable evidence that an excitatory effect on EDA results from hypothalamic control and associated activity in the limbic system and the amygdala [46]. In line with this, neuroimaging technology showed several consistent patterns in the brain, where activations associated with attentional and emotional responses correlated with changes in the electrodermal conductivity.

The EDA signal consists of two main components: skin conductance level (SCL) and skin conductance response (SCR) [46,47]. SCL relates to slowly drifting components; its common properties are a gradually decrease while participants are at rest, and a rapid increase when a new stimulus is presented. SCR relates to spontaneous activations or spikes superimposed on SCL and are triggered by a stimulus [47,48]. In experimental settings, this property was used in attentional tasks with repeated visual stimuli to monitor the emotional and arousal state of participants [49,50]. In contrast, SCL is assumed to be a more reliable indicator for changes occurring with continuous stimuli (i.e., information processing, reading) [46,51]. The EDA is measured in microsiemens units (µS).

The first studies employing EDA for mind wandering detection pointed at induced alternations in dermal properties during episodes of mind wandering. Thus, Blanchard et al. used EDA, skin temperature (ST), and context features (i.e., text, timing, and text difficulty) to build a supervised classification model for automatic mind wandering detection in a learning setting [21]. Using the *Affectiva Q* with a sampling rate of 8 Hz, they recorded data from 70 undergraduate students using a combination of self-caught and probe-caught methods. From EDA and ST, authors calculated the following physiological features: the standardized signal, an approximation of the time-derivative of the signal, the frequency and magnitude from Fast Fourier transformation, the spectral density of the signal and the autocorrelation of the signal at lag 10 (for a comprehensive review see [21]). Then, for the physiological features, the mean, standard deviation, maximum, the ratio of maxima, and ratio of minima were calculated, resulting in 43 features for EDA and ST, respectively. Context features included 11 elements. The model using the combination of features from EDA and contextual features achieved the highest kappa coefficient of 0.15. Kappa ranges from 0 (chance agreement) to 1 (perfect agreement). Following a classification by Landis and Koch [52], a value of 0.14 indicates “slight agreement”. The combination of EDA, ST, and context features achieved a “moderate agreement” with a kappa of 0.22. Unfortunately, their work did not consider a solely EDA-based classification model.

Cheetham and colleagues [22] investigated the feasibility of using only EDA features for the automatic detection of mind wandering in the context of meditation. Using peaks observed from a low-pass filtered EDA signal as a feature, authors reported an area under the curve (AUC) of 0.81. AUC ranges from 0.5 (chance) to 1 (perfect agreement). An AUC of 0.81 is better than Blanchard et al.’s kappa of 0.22, but still not very high. The episodes of mind wandering and resulting physiological changes during meditation might differ, however, from those in tasks requiring higher cognitive functions [9]. Nevertheless, both of these studies did not consider the underlying sub-components of EDA in their analysis.

Based on the aforementioned physiological properties of EDA and validation studies, we hypothesize that spontaneous episodes of mind wandering in a sustained attention task might cause alternations in SCL and/or SCR. Since these physiological dimensions are not accessible for eye-tracking, this work considers the EDA as a complementary mean to boost the performance of automatic mind wandering detection.

## 4. Method

### 4.1. Research Design

To investigate effects of context factors on mind wandering frequency we defined two independent variables—*Text Type* and *Music Type*, and manipulated their levels. We distinguished between three different Music Types: Sad, Happy and No-Music as a control condition. The factor Text Type contained the levels: Computer Science, Psychology and a Random Text Type. Dependent variables Interest, Difficulty, Tiredness, Perceived Mood, Attentional Focus and Type of Thoughts were acquired from behavioral data and are discussed below. We used a repeated-measure design where each participant was involved in all experimental conditions. Thereby, our experiment has a 3 × 3 repeated-measures design (see Table 1). On each level, we used a “triangulation” method by which self-report, behavioral data and physiological measures are used to make an inference about the current mental state [1]. Self-caught reports from participants included timestamps of the experienced episodes of mind wandering, behavioral data included a questionnaire relating to text and self-perception, and physiological measurements included the signals acquired from an eye-tracker and an EDA sensor.

### 4.2. Apparatus

**Figure 1:**
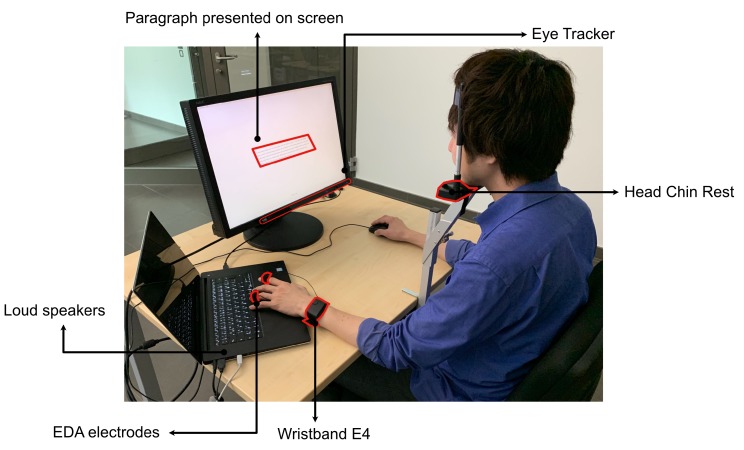
A participant performing the reading task. We collected eye movements using an eye-tracker Tobii 4C, EDA using a wristband Empatica E4, as well as occurrences of mind wandering through self-reports.

For the reading task, we used an NEC MultiSync EA241WM monitor with a resolution of 1920 × 1200 pixels operating at 60 Hz. The distance between the participant and the monitor was fixed at 60 cm (see Figure 1). For the eye-movement recording, a Tobii 4C gaming eye-tracker (https://gaming.tobii.com/tobii-eye-tracker-4c/) with 90 Hz sampling frequency and a scientific license was used. This eye-tracker uses a reflection pattern of NIR (near-infrared) light for the recording of eye movements. To achieve a high accuracy of the eye-tracker and avoid any head movement artifacts, we used a head chin rest. To measure the EDA, we used an Empatica E4 wristband (https://www.empatica.com/research/e4/) with a sampling frequency of 4 Hz. The wristband E4 does not require any calibration. It was placed on the wrist of the non-dominant hand. Since the fingers have the highest density of the sweat glands, we used lead wires (https://store.empatica.com/products/e4-wristband?variant=17719039950953) for index and ring fingers to acquire a higher EDA signal. The lead wires were snapped instead of plated electrodes. The data recording continued over the entire experimental session.

The experiment was programmed in a JavaScript application containing text and audio stimuli, comprehension questions, and questions for behavioral data. The text paragraphs were presented in the centre of the computer screen in a Sans-Serif font, a font size of 18 pixel, and in black color.

### 4.3. Participants

21 graduate and undergraduate students (17 male) with an average age of 25.3 (SD=2.6) years were recruited from the University of Kaiserslautern at the Department of Computer Science via a mailing list. All participants were native German speakers with normal or corrected-to-normal vision. All students had a major in computer science. For the participation in the study, participants were offered either course credits or a 10 euro gift card.

### 4.4. Materials

#### 4.4.1. Reading Material

We employed 12 scientific texts in German language from an online platform (https://www.wissen.de/) that publishes articles in popular science. Since one of the goals of this study was to investigate the influence of semantically relevant information on mind wandering frequency, we deliberately chose eight texts from the categories of Psychology and Computer Science that were assumed to match the personal and academic background of our participants, respectively. Texts in Computer Science included topics from Cryptography, Artificial Intelligence, Storage and Algorithms. Texts in Psychology included topics about Names and Stereotypes, Conscious and Unconscious Self, and Self Portrait. The remaining four texts belonged to the category of Random Text Type and consisted of topics that, to the mind of investigators, could not directly relate to one’s personal or/and academical background. This category included texts about Photosynthesis, Metaphors, Bovine Diseases, and the Australian Football game “Footy”. All the texts had a comparable number of words (Mean=230.4,SD=19.6) and difficulty level (LIX score (https://www.psychometrica.de/lix.html) Mean=57.3,SD=9.3). The average text difficulty corresponded to that of scientific articles. This difficulty level was deliberately chosen in order not to not overtax participants while still providing them with reading materials which would resemble their literature for home works or exams. In the next step, we split each text into four paragraphs of comparable length (mean number of words = 60.9, SD = 6.3). The suitability of this text representation in the context of mind wandering research was demonstrated in studies by Bixler and D’Mello [19], as well as Blanchard and colleagues [21]. Thus, in one experimental session, participants read two texts from each text type, split into four paragraphs presented one by one, resulting in a total of 24 reading segments (2 text × 3 categories × 4 paragraphs). The order of text types was randomized within each participant.

#### 4.4.2. Music Stimuli

Audio stimuli were adopted from Taruffi et al. [9]’s study. The advantage of their stimulus pool is a pretested homogeneity of happy and sad compositions with significant differences to the opposite affective tone. Thus, the set of sad stimuli was rated in their pretest as highly pleasant, slightly arousing, clearly sad, not very happy, and unfamiliar. For the happy conditions, the musical stimuli were rated as highly pleasant, very arousing, clearly happy, not sad, and unfamiliar. We deliberately selected unfamiliar musical stimuli (without vocals) to avoid possible memory effects on mind wandering. The final stimulus set included eight sad and eight happy compositions. They were clipped into 45-s segments and normalized by following the documentation provided by *Audacity* software (https://manual.audacityteam.org/man/dc_offset.html). We randomized sad and happy music segments between paragraphs with respect to their condition, and counterbalanced them with respect to the No-Music conditions. Thus, in one session, participants read 12 paragraphs with and 12 paragraphs without music in the background. The onset and offset of the music playback was synchronized with the beginning and the end of each text paragraph. We set the music volume to a comfortable level and made sure to conduct all experimental sessions under the same conditions. The music played through laptop speakers (see Figure 1).

### 4.5. Procedure

The experiment was performed in accordance with the institutional ethical guidlines of the German Research Centre for Artificial Intelligence. Before starting the experiment, participants were informed about the general aim of the study. After they gave their informed consent, the experiment was started. Participants were also informed that they could withdraw from the study at any point in time. The experimental room was quiet, and we controlled light, temperature and possible noise distractors within it.

We split the experiment into two 45-min sessions. The sessions were run on different days to avoid fatigue and interaction effects between Happy and Sad conditions. Each session belonged either to the Happy or Sad condition depending on the Music Type playing in the background. Before starting the experimental session, participants received the following instruction:

*You will be presented with six different texts split into paragraphs. Each text contains 4 paragraphs. Please read each paragraph as attentively as possible. Ignore possible music in the background. While reading text, your attention might drift from reading to internal thoughts or concerns, which is totally natural. If it happens, please press the space button and focus back on the reading task*.

Before starting the data recording, we asked participants to take a comfortable sitting position which they could maintain for at least the next 10 min. The chin rest was adjusted for each participant individually to prevent any discomfort that might restrict their normal reading behavior. Apart from the chin rest, there were no external devices attached to the head or eyes. To prevent movement artifacts in EDA, we asked participants to rest their hand carrying the wristband on the table. After each sequence of four paragraphs (one complete text), participants could take a short break. During the breaks they were reminded to report mind wandering in the reading task. After each break, the eye-tracker was re-calibrated using a standard 5-point calibration method. To ensure participants were reading attentively, each paragraph was followed by a comprehension question with one possible answer, “yes” or “no”. Next, the participants were asked to rate each paragraph on Interest, Difficulty, Tiredness, Personal/Academic Relevance, Perceived Mood (Sadness and Happiness), Attentional Focus and Type of Thoughts (task-relevant or task-irrelevant) (the questionnaire was adopted with changes from Giambra and Grodsky [33]):

Q1. How interesting did you find the last paragraph? (0%: not interesting at all, 100%: very interesting)
Q2. How difficult did you find the last paragraph? (0%: not difficult at all, 100%: very difficult)
Q3. How tired did you feel while reading the last paragraph? (0%: not tired at all, 100%: very tired)
Q4. What was your level of happiness while reading the last paragraph? (0%: not happy at all, 100%: very happy)
Q5. What was your level of sadness while reading the last paragraph? (0%: not sad at all, 100%: very sad)
Q6. Did the context of the last paragraph match your academical or personal background?(0%: did not match at all, 100%: completely matched)
Q7. While reading the last paragraph, where was your attention focused? (-5: completely absorbed in own thoughts, +5: completely focused on the text)

For the questions *Q1–Q6*, we used 5-point rating scales with step sizes of 20%. *Q7*—Attentional Focus was used as an additional retrospective ES [6] to enhance the determination of the experienced type of thoughts. Thus, if participants rated their attentional focus with less than +3 (step size: 1 point), two additional questions (*Q8* and *Q9*) were automatically displayed:

Q8. While reading the last paragraph, did you have some text-related thoughts? (yes/no)
Q9. *While reading the last paragraph, did you have some text irrelevant thoughts (i.e., personal worries, future planning, dreams, thoughts about your relatives or friends)? (yes/no)*.

Except for the variables Sadness and Happiness, which are not further analyzed in this study, the entire set of questions is considered further as *behavioral data*. Additionally, Reading Duration (time spent to read one paragraph) was recorded within each participant and considered as a behavioral feature.

## 5. Analysis of Behavioral Data and Results

To investigate possible effects of music and text type on mind wandering, we tested within-subject contrast effects using a two-way ANOVA (analysis of variance) for a two-factorial repeated-measure design. For this purpose, the mean values of each variable underwent a planned pairwise comparison as outlined in Table 1. All reported F-values for the main and contrast effects are Greenhouse-Geisser corrected. All significant tests are reported. To analyze simple effects within each factor, we employed Helmert contrasts where we first compared the two non-control conditions (Happy and Sad Music, Computer Science and Psychology Text Types) and then compared the mean of those two conditions with the control (No-Music, Random Text Type). One participant was excluded from the final analysis after he admitted to have misunderstood the experimental instructions. The final sample size used for the statistical analysis included 20 participants.

### 5.1. Personal/Academic Relevance

To confirm the validity of our assumptions on personal/academic text relevance (PAR), the effect of Text Type on perceived PAR was examined. An ANOVA yielded a significant main effect of Text Type on PAR (see Table A1 in Appendix A). The contrast analysis showed that the paragraphs from Computer Science (*mean* = 52.08, *SD* = 29.08) were rated as significantly more relevant than those from Psychology (*mean* = 20.98, *SD* = 25.29). The relevance of the paragraphs from Random Text Type (*mean* = 6.17, *SD* = 13.74) was perceived as significantly lower than those from Computer Science and Psychology (see Table A1 in Appendix A).

### 5.2. Mind Wandering

Table 2 represents the average mind wandering frequency within a particular condition. The main effect of Music Type on mind wandering was significant (see Table A1 in Appendix A). Although there was no significant contrast effect between Happy and Sad Music on the mind wandering frequency, participants were mind wandering significantly more frequently in conditions with music (Happy and Sad combined) comparing to the No-Music condition (see Table A1 in Appendix A). We could not observe any significant effects of Text Type on the mind wandering frequency. However, there was a significant interaction effect between Music Type and Text Type on mind wandering: while reading texts in Computer Science and listening to Sad Music, participants experienced more frequent mind wandering compared to the conditions with the same Music Type but texts in Psychology or Random Topic. The opposite effect was observed for Random Text Type: while listening to Happy Music participants reported episodes of mind wandering significantly more frequently compared to Sad Music and the control condition.

### 5.3. Task-Related Thoughts/ Task-Unrelated Thoughts

Because the variables *Q8* and *Q9* were coded binarily, we used the arcsine transformation of TRTs and TUTs to meet ANOVA requirements. A significant main effect of Text Type on TRTs was found. The contrast analysis showed that while reading paragraphs from Random Topics participants experienced TRTs significantly more frequently (see Table 2) compared to paragraphs from Computer Science and Psychology (see Table A1 in Appendix A). There was no significant main effect of Music Type on TRTs and no interaction. No significant main effects or interactions were observed for TUTs.

### 5.4. Correlation Analysis

In the next step, we ran a multivariate Pearson’s R correlation test to explore possible dependencies between variables. Here, we report only significant correlations with the Pearson’s *r* coefficient ≥ ± 0.2.

There was a highly significant, moderate and negative correlation between mind wandering and Attentional Focus (*r* = −0.38, *p* < 0.001). Attentional Focus had a statistically significant but low to moderate, positive correlation with Personal/Academic Relevance (r=0.25,p<0.001). A significant, moderate, positive correlation was observed between Attentional Focus and Interest (r=0.34,p<0.001). Moderate to low, negative correlations were observed between Attentional Focus and Tiredness (r=−0.35,p<0.001), and Difficulty (r=−0.24,p<0.001). There were highly significant, moderate to low, negative correlations between Attention Focus and TRTs (*r* = -0.40, *p* < 0.001), and TUTs (r=−0.20,p<0.001), respectively. Personal/Academic Relevance was found to have a highly significant, positive correlation with Interest (r=0.54,p<0.001). We observed a statistically significant, moderate and negative correlation between TRTs and Interest ( r=−0.40,p<0.001). Statistically significant, small, positive correlations were present between TRTs and Tiredness and Difficulty with (r=0.25,p<0.001) and (r=0.22,p<0.001), respectively.

## 6. Mind Wandering Detection

The dataset collected from participants was used to engineer features and to build machine learning models for detecting events of mind wandering. The pipeline for data pre-processing, feature engineering and model building is carried out in Python using the libraries *numpy*, *pandas*, *scipy*, *sklearn* and *matplotlib*.

Beside the features acquired from the sensors (see Section 6.1 and Section 6.2, we also included behavioral features in classification models, namely Reading Duration, Interest, Difficulty, and Tiredness.

### 6.1. Eye-Tracker Feature Extraction

**Figure 2:**
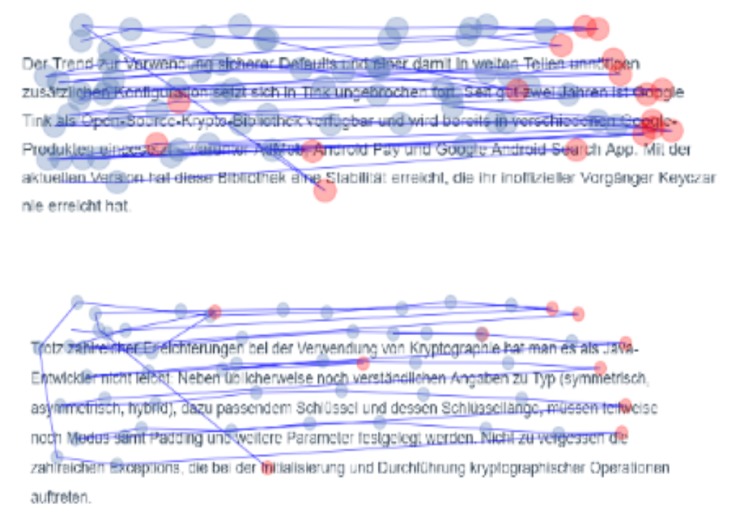
Eye movements during the reading task (fixation points in blue and regression points in red). **Top**: Paragraph with reported mind wandering, **bottom**: paragraph with focused reading behaviour.

We used the Tobii Pro software to extract the raw eye data and information about the pupil diameter. We clustered the raw gaze points into fixations using the Dispersion-Threshold Identification algorithm [53]. Further, we pre-processed the extracted eye fixations by removing those points which were outside the reading area. Eye-tracking data from 59 paragraphs with an insufficient eye-tracker accuracy distributed across participants were also excluded from the classification tasks. As a result, the final data for detecting mind wandering included eye-tracking data from 871 paragraphs. Finally, we visualized the eye movements and compared paragraphs with respect to the presence or absence of reported mind wanderings (see Figure 2).

For each paragraph, we extracted low-level fixation related features, such as the mean fixation points etc., from the obtained fixation points. We further computed saccades from the gaze data and computed saccade-related features, such as mean saccade length and number of regression etc., for each paragraph. Pupil size was also included in our feature set. Detailed description of each included feature is provided in Table 3. In total, 19 eye movement features were calculated.

### 6.2. EDA Feature Extraction

To extract EDA features, we used the convex optimization approach proposed by Greco and colleagues [44]. This method postulates that EDA can be described as a function of three components: a slow tonic, the output of convolution between an infinite impulse response function (IIR) and a sparse non-negative sudomotor nerve activity (SNMA) phasic driver. The tonic component corresponds to a slowly changing component of EDA. It is related to general information about psycho-physiological state [44], and can also be linked to the attentional state [54]. The phasic component is associated with the short-term changes in EDA as a reaction to stimuli. The phasic component includes higher frequency components, in particular a phasic driver.

**Figure A1:**
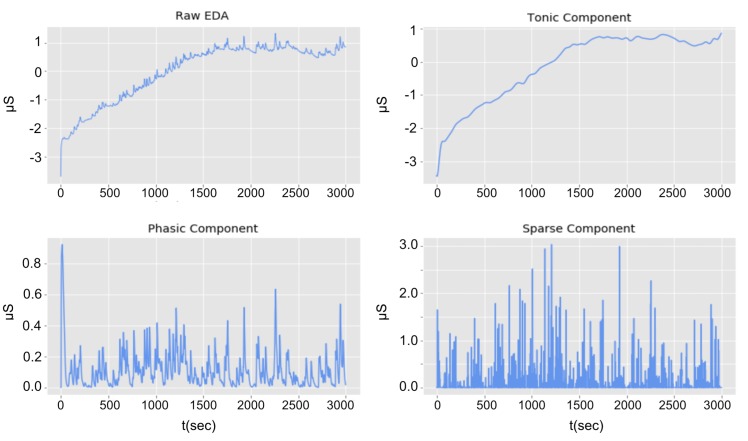
Example of Convex Optimization Approach for EDA Decomposition. (**Top left**): EDA signal (z-score). (**Top right**): Tonic component. (**Bottom left**): Phasic component. (**Bottom right**): Sparse component (phasic driver).

In accordance with the guideline on convex optimization [44] for EDA decomposition, we first applied a z-standardization of the acquired signal within each subject to accommodate the usually high intersubject variability of EDA signals [45,46,55]. Figure A1 represents the resulting components from the acquired EDA signal after convex optimization. In the next step, we calculated statistical features. For the raw z-standardized signal, the tonic, and the sparse components, we computed *mean*, *standard deviation*, *minimum*, and *maximum* values. For the sparse component, we calculated *mean* and *standard deviation* as well as *minimum* and *maximum values* for the *peak amplitude*, the *number of peaks within one paragraph* and the *number of peaks above 1 µS* [56]. The 59 paragraphs excluded because of the low eye-tracking accuracy were also not considered for EDA-based model building and evaluation. The EDA feature set included 18 features.

### 6.3. Model Building

We used the extracted feature sets to build user-independent classification models that detect episodes of mind wandering while reading. Each reading paragraph was labelled based on participants’ reports (self-caught method): “1” for the presence of mind wandering and “0” for no mind wandering, which served as ground truth for the classification models. The data from one participant were excluded from model building since he had not reported any episodes of mind wandering. Thus, the data from 19 participants were used for model building.

We chose three different machine-learning algorithms for mind wandering detection: Logistic Regression (which served as a baseline classifier), Support Vector Machine (SVM), and Random Forest.

Before being fed into the classification models, training data were processed in two ways: First, we applied feature standardization using *StandardScaler* to achieve equal scaling among input features. The standardization was performed separately for the training and testing dataset within each fold. Second, the class “1”, which represented the presence of mind wandering, was only reported in 15.8% of all paragraphs. Hence, after defining training and testing datasets, we over-sampled the class “1” using the synthetic minority over-sampling technique (SMOTE) [57]. The appropriateness of this sampling technique for the particular classification problem was demonstrated in a study by Bixler and D’Mello [14]. It must be emphasized that we applied SMOTE to the training dataset only. For Logistic Regression we used the leave-one-participant-out cross-validation method, where the data of one participant were withheld for testing, and the remaining data were used to train the model. This procedure was repeated 19 times (based on the total number of participants).

### 6.4. Model Validation

To avoid the risk of overfitting and to ensure that the Random forest and SVM-based models were built using the best hyper-parameters, we performed a nested cross-validation procedure [58]. The outer loop in nested cross-validation is for model assessment, and the inner loop is for hyper-parameter tuning. We used leave-one-participant-out cross-validation in the outer loop for the model assessment. In the inner loop, we used the Scikit-Learn’s GridSearchCV (https://scikit-learn.org/stable/modules/generated/sklearn.model_selection.GridSearchCV.html) method to define a grid of hyperparameters and performed 3-fold cross-validation to tune the hyperparameters. The SVM and the Random Forest models were then built on the outer loop training data using the best-selected hyper-parameter combination, and the model evaluation was performed on the test fold (one participant). The nested cross-validation procedure prevents the model from biasing to the dataset, which would result in overly-optimistic scores. This artifact is described in more detail by Cawley and Talbot [58] and would otherwise occur when using a single (not-nested) cross validation.

As the dataset for the classification of mind wandering was imbalanced (mind wandering occured in 15.8 % of paragraphs), we reported four evaluation measures. First is the $F_{1}$-Score (weighted average), which is essentially the weighted harmonic mean of the precision and recall, and is commonly used in the literature to report the classification accuracy of an imbalanced dataset [59]. Therefore, we further report our results using only $F_{1}$-Scores. Second is the area under the Receiver Operating Characteristic curve (AUC), which depicts the capability of the classifier to distinguish between classes. We also report the Cohen’s Kappa and Accuracy. For these two evaluation metrics the best classification accuracy is 1.0 and the worst is 0.0. The latter three metrics are used in the section *Discussion* to provide a comparison of the obtained results with related studies.

### 6.5. Results

#### 6.5.1. Comparison of Machine Learning Algorithms

The performance of the three machine learning classifiers is first compared along the task of detecting mind wandering during reading. As seen in Table 4, compared with Random Forest and SVM, Logistic Regression shows the lowest classification accuracy, the reason being that Logistic Regression does not work well when a correlation is found between features [60]. As our dataset is highly complex and the features extracted using different sensors may correlate, Logistic Regression was not suited well for our task. The Random Forest-based model has the highest $F_{1}$-Score for the entire feature set. But the performance of this classifier is dependent on the hyperparameters chosen (number of trees, maximum depth etc.) [61]. Lastly, SVM shows similar, yet slightly lower $F_{1}$-Scores for mind wandering detection. Compared to Random Forest, it works better for the model using several feature sets (Eye + EDA + Behavior). With an appropriate choice of the kernel, SVM is not sensitive to the correlation between features, and generally performs well for a multivariate dataset [60].

Because Random Forest showed the highest $F_{1}$ Score of all the models, in the following we report the classification results from the Random-Forest-based classification models, except where other classification models or metrics are explicitly mentioned.

#### 6.5.2. Model Performance on Combined Features

**Figure 3:**
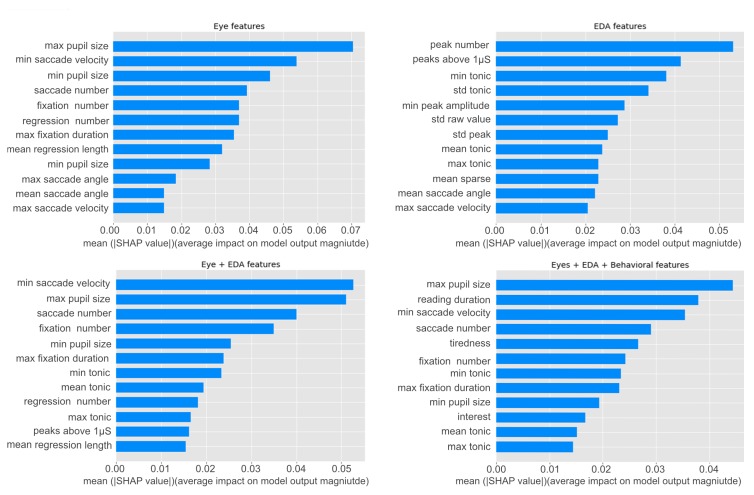
Feature importance graph for the Random Forest classification models usig the SHAP method. **Top left**: Eye-based model. **Top right**: EDA-based model. **Bottom left**: Eye and EDA-based model. **Bottom right**: Sensory and Behavior-based model.

To analyze how different features influence the performance of the Random Forest model, we first built our model using all the combined features of the sensors and behavioral data. Followed by this, we investigated the feasibility of each sensor for mind wandering detection by building separate models using only eye and only EDA features. We further investigated the feature importance of each model tuned to the best hyperparameters using the SHAP (Shapley Additive Explanations) [62] method. This method shows the contribution of each feature by comparing what a machine learning model predicts with and without that feature. The SHAP values reflecting the importance of each feature for the random forest model are shown in Figure 3.

The EDA-based model achieved an $F_{1}$-Score of 0.78 (see Table 4). In this model, the *number of peaks* had the highest contribution for mind wandering prediction. The number of *peaks above 1 µS* and the *min value* of the *tonic component* had nearly the same feature importance followed by the *standard deviation* of the *tonic component*, and the *peak standard deviation* (see Figure 3). The eye-based model was observed to have a slightly higher classification accuracy than the EDA-based model with an $F_{1}$-Score of 0.80 (see Table 4). Low-level eye features like *pupil size*, *saccade velocity*, *number of fixations* and *fixation duration* were considered to be the most important for mind wandering detection as shown in Figure 3. We further noted that the combination of the eye and EDA features led to an improvement of the classification accuracy by 3%. Thus, this model achieved an $F_{1}$-Score of 0.83 and resulted in the best classification model (see Table 4). This increase in the classification accuracy could occur because both sensors complement each other by capturing different dimensions of the user’s cognitive state, improving the overall classification accuracy.

Lastly, we found that behavioral data combined with the sensor features (eye + EDA + behavioral) led to no further than a moderate classification improvement for mind wandering detection using SVM ($F_{1}$-Score: 0.82) (see Table 4). For this model, *saccade velocity*, *reading duration* and *pupil size* had the highest importance for mind wandering detection (see Figure 3), followed by *tiredness* and fixation related features, and, finally, the *minimum* value of the *tonic component*. For the Random Forest no further improvement over the eye and EDA-based model was observed (see Table 4).

## 7. Discussion

### 7.1. Text, Music and Mind Wandering

Our results show a statistically significant interaction effect between Text and Music Type on the frequency of mind wandering. Sad Music promoted mind wandering when participants read paragraphs about a topic that was academically relevant to them (Computer Science), compared to the other text types and compared to Happy or no music. Sad music has previously been proposed to lead to lower emotional arousal, which in turn may result in a higher mind wandering frequency [9].

For paragraphs from Random text types, an opposite effect was observed. Participants reported mind wandering more frequently while listening to Happy Music. This leads us to conclude that happy music was perceived as more disruptive, especially if participants did not have prior knowledge of the topic. The combination of disruptive music and new information could lead to a failure in the construction of a situation model for reading paragraphs, resulting in a higher frequency of mind wandering [63,64]. We also found that the presence of music in the reading task significantly increased the frequency of mind wandering compared with reading without music. These findings are consistent with previous studies that observed a higher mind wandering frequency in conditions with unfamiliar music [38,39]. Our assumption that mind wandering occurs more frequently when reading texts of personal or academic relevance was not confirmed. One potential reason for this outcome could be that paragraph length was insufficient to generate the full semantic effect on mind wandering [65]. Other studies investigating the relationship between semantics and mind wandering used a longer text presentation [32].

The contrast analysis revealed a significantly higher number of task-related thoughts while reading the Random text type. Since the texts from Random text type were chosen to avoid any matching personal or academic background of the participants, we assume that these TRTs were related to low prior knowledge. Our assumption is consistent with the study of Kane and colleagues [31], who showed that at the early learning stages students tend to have meta-cognitive thoughts about how well they understand the new material. Interestingly, unlike in studies of Kane et al. [31] and Jing et al. [23] that reported a moderate to strong, positive correlation between situational interest and mind wandering, we obtained a significant low to moderate, negative correlation between TRTs and Interest. This difference could occur because of a discrepancy in the definition of Interest: while in our study it was attached to the thematic content of the paragraphs, other studies defined it in terms of “interest in the lecture” and “utility of the lecture” [31].

Although behavioral data do not allow us to pinpoint the exact content of reported TRTs, we can make several assumptions about their direction. We hypothesize that while reading new information, participants were able to shift their attention to memory to reconcile the new information with some existing prior knowledge, as discussed by Benedek et al. [42]. We suppose that TRTs at a low level of interest could contain judgmental or emotional components pertaining to the text or task perception. Our assumption is partly supported to the study by Faber and D’Mello [32], who recorded and categorized different types of internal thoughts; however, without considering a particular interest level. The most typical TRTs reported in their study were: “If the experimenters chose this text because it is very boring and they want us to zone out a lot,” “If I am going to be quizzed on this,” “I was wondering how much time was left”.

### 7.2. Automatic Detection of Mind Wandering

We demonstrate the feasibility of combining EDA sensor data and eye movements recorded by a low-cost eye-tracker for automatic, user-independent mind wandering detection.

The EDA feature-based SVM model achieved an $F_{1}$-Score of 0.76 and a kappa coefficient of 0.26. Thereby, using only EDA features this model outperformed the model by Blanchard et al. [21] (EDA and contextual features) by 11 kappa points. This improvement can be explained through the different approach used for EDA signal processing.

The eye-tracker-based model achieved a high detection performance with an $F_{1}$-Score of 0.80, outperforming the reported results of the comparable studies by Hutt et al. ( an $F_{1}$-Scores of 0.59 [43], and 0.47 [28]); showing the sufficient sensitivity of a single sensor for mind wandering detection. The results of the SHAP method resemble the prior studies [16,41,42]: statistical features of pupil size, saccades and fixations were found to have the highest impact for the detection of mind wandering.

As we assumed earlier, electrodermal activity is related to emotional arousal triggered by episodes of mind wandering, whereas eye movements are related to the visual attention of learners. The model based on these two feature sets achieved the highest classification accuracy for mind wandering and outperformed the eye-based model by 3%. At first glance, this improvement can appear rather moderate. It points, however, at the general possibilities for combining features acquired from an eye-tracker and an EDA sensor. Moreover, combining the two sensors can be useful because they operate in different time frames: whereas changes in eye-movements caused by cognitive processes can be observed on a millisecond level [66], the alternations in EDA can be visible up to three seconds after a stimulus occurred [46]. We therefore presume that when reading longer texts, the combination of EDA and eye-tracker may result in still higher classification accuracy because the shortness of the paragraphs may have led us to miss some information in the EDA if the mind-wandering occurred towards the end of the paragraph.

The further inclusion of behavioral features did not show a remarkable contribution for the detection of mind wandering, albeit reading duration strongly contributed to the classification accuracy. This result shows that for an automatic mind wandering detector the sensory data are sufficient, eliminating the necessity to use additional behavioral questionnaires.

### 7.3. Application Scenario

Being able to automatically detect episodes of mind wandering on an individual level allows for a range of applications. In a classroom setting, mind wandering quantification can provide feedback to learners on how attentive and immersed they were in the learning material so far. The parts in the learning material associated with mind wandering can subsequently be visualized. Sharing these insights with instructors and those who create course materials can help to improve content and delivery. Moreover, the detection of mind wandering can be especially relevant to groups of learners with attentional disorders such as ADHD (attention deficit hyperactivity disorder) [67], providing a gentle support to make learners aware of their learning and attentional states.

### 7.4. Limitation and Future Work

The work presented here has several limitations. First, we used the self-caught method to collect episodes of mind wandering. The accuracy of this method strongly relies on participants’ ability to track their thought process and to report any episode of mind wandering. Because of the experimental design, we decided not to include an additional probe-caught method to avoid presenting another potentially distracting stimulus. Moreover, the proposed method is an offline mind wandering detection technique. Because of the low sampling frequency of EDA sensors (4 Hz) and the physiological time-course of electrodermal responses (up to 3 s response delay to stimuli), a moment-to-moment detection of mind wandering using the particular combination of sensors is very challenging. However, it is possible to provide some feedback to learners, for example, how attentive they have been working with a learning material within the last 30 s.

It is also important to emphasize, that for EDA we did not deploy spectral analysis [47] for the feature calculation. Our classification model based on the statistical features for the EDA components outperformed the model from the related study using features based on spectral analysis [21]. However, the deployment of spectral analysis in future work could provide more insights into the alternations in the EDA components during mind wandering, as well as an additional increase in classification accuracy.

In addition, in this study we used a binary classification to detect the presence or absence of mind wandering. In future research, it is necessary to investigate the possibilities to identify the particular type of mind wandering (TRT or TUT). This distinction is important from a pedagogical point of view, where mind wandering is often associated with task-unrelated thoughts that are part of metacognition about unfamiliar, demanding, or boring tasks and may actually contribute to the ultimate learning success. This aspect should be considered while building attention-aware systems.

Finally, the feasibility of EDA and eye-tracking as a multimodal sensor setup for mind wandering detection was explored in a controlled experiment. Therefore, it will be essential to verify these results in a real classroom setting.

## 8. Conclusions

Even though we did not find any effect of semantic relevance on mind wandering frequency, we showed that music (no matter whether happy or sad) promoted the occurrence of mind wandering. We showed that the EDA sensor has a high potential as a single sensor in mind wandering detection. Moreover, the combination of electrodermal activity and eye-tracking features improved the classification accuracy of mind wandering. Finally, the scalability and cost of these devices makes the proposed technique applicable for classroom usage.

## Figures and Tables

**Table 1:** Experimental Design: Experimental and Control Conditions with a Total Sample Size.

3 × 3		Music Type		
		Sad	Happy	No-Music
	Psychology	80	80	160
**Text Type**	Computer Science	80	80	160
	Random Topic	80	80	160

**Table 2:** Descriptive Statistics.

Condition	Mind Wandering M(SD)	TRTs M(SD)
Sad Computer Science	0.34(0.38)	0.77(0.88)
Sad Psychology	0.18(0.26)	0.87(0.98)
Sad Random Topic	0.19(0.27)	0.91(1.05)
Happy Computer Science	0.22(0.35)	0.47(0.67)
Happy Psychology	0.25(0.42)	0.53(0.76)
Happy Random Topic	0.39(0.52)	1.01(1.06)
Baseline Computer Science	0.12(0.18)	0.60(0.72)
Baseline Psychology	0.08(0.11)	0.60(0.75)
Baseline Random Topic	0.15(0.14)	0.87(0.92)

**Table 3:** Eye tracker features. For all features mean was calculated, for bolded features min, max values were additionally calculated.

Features	Description
**Fixation Duration**	Duration of a fixation point in milliseconds
**Pupil size**	Diameter of pupil in pixels (z-score)
**Saccade length**	Distance in pixels between two subsequent fixations
**Saccade velocity**	Transition between two subsequent fixations in milliseconds
**Saccade angle**	Angle in radians between *x* axis and the ray to the point (*x*, *y*)
**Regression length**	Backward transition between two fixation points in pixels
Number of regressions	Total number of regressions within one paragraph
Number of fixations	Total number of fixation points within one paragraph
Number of saccades	Total number of saccades within one paragraph

**Table 4:** In this table we report the results for different classifiers and feature sets. The numbers are reported in percentages. As seen in the table, all random forest-based classifiers achieved the highest $F_{1}$-Score. The combination of Eye and EDA Features achieved the highest classification accuracy.

Classifier	Feature Type	Kappa	Accuracy	AUC	$F_{1}$-Score	Presicion	Recall
	Eye	0.25(0.21)	0.72(0.14)	0.70(0.12)	0.75(0.12)	0.85(0.11)	0.72(0.15)
**Logistic**	EDA	0.23(0.28)	0.70(0.17)	0.66(0.19)	0.73(0.16)	0.84(0.12)	0.70(0.18)
**Regression**	Eye + EDA	0.26(0.22)	0.73(0.13)	0.71(0.16)	0.76(0.11)	0.85(0.10)	0.73(0.13)
	Eye + EDA + Behavior	0.31(0.27)	0.76(0.13)	0.72(0.17)	0.79(0.10)	0.86(0.09)	0.76(0.13)
	Eye	0.25(0.21)	0.80(0.09)	0.66(0.12)	0.80(0.09)	0.83(0.12)	0.80(0.09)
**Random**	EDA	0.15(0.15)	0.83(0.08)	0.62(0.13)	0.78(0.08)	0.82(0.09)	0.77(0.08)
**Forest**	Eye + EDA	0.29(0.27)	0.83(0.08)	0.65(0.16)	0.83(0.08)	0.84(0.11)	0.83(0.08)
	Eye + EDA + Behavior	0.31(0.27)	0.76(0.13)	0.69(0.15)	0.82(0.09)	0.86(0.10)	0.82(0.10)
	Eye	0.26(0.24)	0.78(0.13)	0.68(0.13)	0.78(0.11)	0.85(0.10)	0.77(0.13)
**SVM**	EDA	0.26(0.23)	0.73(0.14)	0.67(0.16)	0.76(0.12)	0.86(0.09)	0.73(0.14)
	Eye + EDA	0.37(0.27)	0.79(0.15)	0.73(0.17)	0.80(0.12)	0.87(0.10)	0.79(0.15)
	Eye + EDA + Behavior	0.41(0.28)	0.80(0.14)	0.77(0.14)	0.82(0.07)	0.88(0.09)	0.80(0.14)

## Appendix A

**Table A1:** Analysis of Variance. Bold font represents main and interaction effects.

Source	df	F	*p*
**PAR**			
**Text Type**	1.9	79.7 **	0.01
C vs P	1.0	64.3 **	0.01
RT vs (C+P)	1.0	94.3 **	0.01
**MW**			
**Music Type**	1.6	3.5 *	0.05
S vs H	1.0	0.4	0.52
(S + H) vs NM	1.0	10.3 **	0.01
**Text Type**	1.7	1.2	0.30
**Text × Music**	2.7	2.8 *	0.05
S CS vs (S RT + S P)	1.0	6.6 *	0.02
H RT vs (H CS + H P)	1.0	5.1 *	0.04
**TRTs**			
**Text Type**	1.7	4.3 *	0.03
R vs (C + P)	1.0	6.1 *	0.02
**Music Type**	1.7	4.3	0.11
**Text × Music**	2.8	1.2	0.31
**TUTs**			
**Text Type**	1.9	0.1	0.87
**Music Type**	1.4	0.6	0.48
**Text × Music**	3.1	0.8	0.53

PAR: Personal/Academic Relevance. MW: Mind Wandering. NM: No-Music, S: Sad, H: Happy. C: Computer Science. P: Psychology. RT: Random Topic. * *p* < 0.05, ** *p* < 0.01.
